# Function of Polyamines in Regulating Cell Cycle Progression of Cultured Silkworm Cells

**DOI:** 10.3390/insects12070624

**Published:** 2021-07-08

**Authors:** Li Chang, Zhiqing Li, Hao Guo, Wenchang Zhang, Weiqun Lan, Jue Wang, Guanwang Shen, Qingyou Xia, Ping Zhao

**Affiliations:** 1 State Key Laboratory of Silkworm Genome Biology, Biological Science Research Center, Southwest University, Chongqing 400715, China; cl17783721226@163.com (L.C.); guohaosf001@163.com (H.G.); zwczmu@163.com (W.Z.); sparkle2021lwq@163.com (W.L.); gwshen@swu.edu.cn (G.S.); xiaqy@swu.edu.cn (Q.X.); zhaop@swu.edu.cn (P.Z.); 2 Chongqing Key Laboratory of Sericultural Science, Chongqing Engineering and Technology Research Center for Novel Silk Materials, Southwest University, Chongqing 400715, China; 3 College of Sericulture, Textile and Biomass Sciences, Southwest University, Chongqing 400715, China; 15123540502@163.com

**Keywords:** *Bombyx mori*, polyamines, cell cycle progression

## Abstract

**Simple Summary:**

The mechanism of the polyamine pathway in the lepidopteran silkworm is largely unknown. In the current study, we aimed to characterize the function of polyamines and polyamine pathway genes in silkworm cells as a regulator of cell cycle progression. For the first time, we identified the homologous genes of the polyamine pathway in silkworm, and analyzed their expression characteristics in different tissues and their subcellular localizations in cultured silkworm cells. We measured the abundant levels of polyamines in silkworm cells by HPLC analysis. We found that exogenous supplementation of spermidine in cells promoted DNA replication and cell cycle progression and, in contrast, treatment with polyamine biosynthesis inhibitors DFMO and MGBG prevented DNA replication and cell cycle progression. Indeed, the mechanism studies indicated that spermidine increased the expression of cell cycle-related genes, whereas this increase could be abolished by treatment with inhibitors. Taken together, our findings highlight that appropriate levels of polyamines have beneficial effects on the progression of the cell cycle by regulating cell cycle genes in silkworm.

**Abstract:**

Background: Putrescine, spermidine, and spermine are polyamines that are ubiquitously distributed in prokaryotic and eukaryotic cells, which play important roles in cell proliferation and differentiation. Methods: We investigated the expression profiles of polyamine pathway genes by qRT-PCR in different tissues of the lepidopteran silkworm. The polyamine levels in cultured silkworm cells were measured by HPLC. Spermidine and polyamine biosynthetic inhibitors were used for treating the cultured silkworm cells in order to clarify their effects on cell cycle progression. Results: We identified the anabolic and catabolic enzymes that are involved in the polyamine biosynthetic pathway in silkworm. Transcriptional expression showed at least seven genes that were expressed in different silkworm tissues. Treatments of the cultured silkworm cells with spermidine or inhibitor mixtures of DFMO and MGBG induced or inhibited the expression of cell cycle-related genes, respectively, and thus led to changed progression of the cell cycle. Conclusions: The present study is the first to identify the polyamine pathway genes and to demonstrate the roles of polyamines on cell cycle progression via regulation of the expression of cell cycle genes in silkworm.

## 1. Introduction

Polyamines are low-molecular-weight polycationic aliphatic amines derived from the metabolism of arginine, which mainly include putrescine, spermidine, and spermine, and are found in prokaryotic and eukaryotic cells [1,2]. Due to their polycationic nature, polyamines are fully protonated under physiological pH and ionic strength conditions. Therefore, they can easily interact with negatively charged macromolecules, such as DNA, RNA, ATP, phospholipids, or proteins [3,4,5], and thereby participate in a number of biological processes, such as modulation of gene expression and enzyme activities, activation of DNA synthesis, transcriptional processes, and regulation of cell proliferation and differentiation [6,7,8].

The biosynthesis of polyamines is regulated by multiple feedback loops that maintain the homeostasis of their metabolites in cells [7]. It begins with the conversion of putrescine from the amino acid ornithine by the enzyme ornithine decarboxylase (ODC), a rate-limiting enzyme in polyamine synthesis [9,10]. Putrescine is sequentially converted to the higher polyamines spermidine and spermine via spermidine synthase (SPDS) and spermine synthase (SPMS), respectively, together with S-adenosyl-methionine decarboxylase (SAMDC), the second rate-limiting enzyme [7,11]. The synthesis of spermine and spermidine is reversible by the action of spermine oxidase (SMO) and acetylpolyamine oxidase (APAO), and requires the spermidine/spermine acetyltransferase (SSAT) enzyme [7].

In addition to the de novo biosynthesis of polyamines in cells, polyamines are also natural components of our diets. For example, spermidine and spermine are found in foods of both plant and animal origin, such as soybeans, mushrooms, cowpeas, fruits, shellfish, and meat products. These dietary polyamines can be absorbed by our gut and transported into different tissue cells, and further regulate the intracellular homeostasis of polyamines [12].

It has been shown that a deficiency of polyamines in cells delays cell cycle progression, with most cells arrest at the G1 to S phase, and the rate of DNA synthesis is also decreased [13,14]. The basic functions of polyamines have been related to cellular DNA protection against exogenous agents and radiation injuries [15,16]. To date, studies on polyamines have mainly focused on spermidine. The evidence has shown that spermidine can promote the growth and development of cells, as well as accelerate the occurrence of autophagy and delay aging [17]. The addition of spermidine in micromolar concentrations into diets has been found to promote human hair growth, and prolong the mean lifespan of *Drosophila melanogaster* and *Caenorhabditis elegans* [17,18]. Additionally, excessive application of spermidine can cause serious diseases, such as respiratory symptoms, nephrotoxicity, and even cancers [19,20].

To investigate the functions of polyamines in cells, a number of inhibitors have been developed to disturb the biosynthesis and homeostasis of polyamines. Among of these inhibitors, difluoromethylornithine (DFMO) and methylglyoxal-bis(guanylhydrazone) (MGBG) are two critical inhibitors that can specifically interfere with the key enzyme activities of ODC and SAMDC, respectively [21,22,23,24]. A combination treatment with DFMO and MGBG can be used to produce a profound anti-proliferative effect and the cell cycle of the cells is arrested at the G1 to S phase, which has demonstrated significant therapeutic effects [7,13].

The silkworm *Bombyx mori* is an economically important insect for silk production and a lepidopteran model for investigating gene functions [25,26]. Although previous studies have demonstrated the positive effects of spermidine supplementation on the development of silk glands, as well as economic parameters [27,28], the enzymes involved in the polyamine pathway and the function of polyamines in the silkworm remain unexplored. Therefore, in the present study, we identified the genes in the polyamine pathway, detected their expression characteristics in different tissues, and analyzed their subcellular localization in cultured silkworm cells. We also determined the levels of various polyamines in cultured silkworm cells by HPLC, and further investigated the cellular function of polyamines by exogenous addition of spermidine and polyamine biosynthetic inhibitors.

## 2. Materials and Methods

### 2.1. Cell Culture

The BmN cell line derived from the silkworm ovary was used in the present work [29]. BmN cells were grown in TC-100 medium (Sigma, St. Louis, MO, USA) supplemented with 10% fetal bovine serum (FBS, Hyclone, Logan, UT, USA) and penicillin-streptomycin (Thermo Fisher Scientific, Waltham, MA, USA) at a temperature of 27 °C. Spermidine was purchased from Sigma (St. Louis, MO, USA), DFMO inhibitor was obtained from Tocris Bioscience (Bristol, UK), and MGBG inhibitor was obtained from Beijing Zongheng Technology Co, Ltd. (Beijing, China).

### 2.2. Identification of Polyamine Pathway Genes in Silkworm

**Figure 1:**
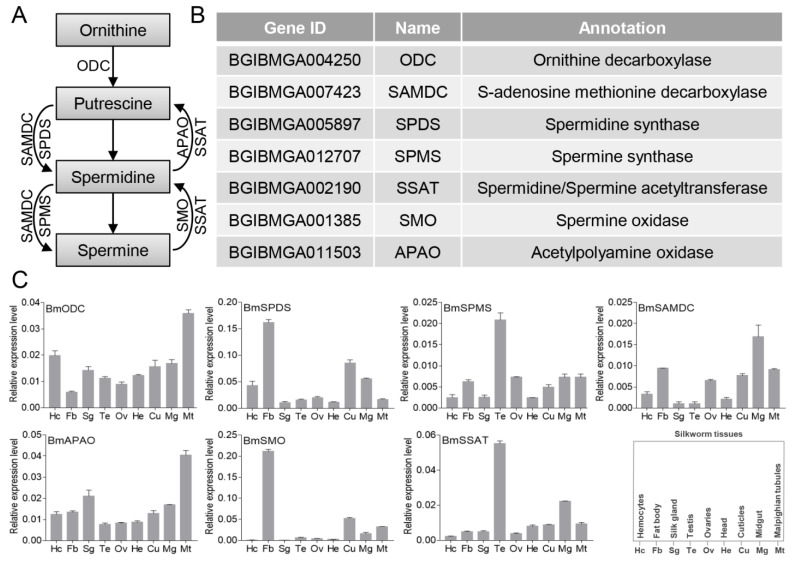
Identification and expression profiles of polyamine pathway genes in the silkworm. (**A**) A schematic representation of the polyamine biosynthesis pathway. (**B**) Identification and annotation of polyamine pathway genes. (**C**) Tissue expression profile of polyamine pathway genes in the silkworm.

The annotated polyamine pathway genes from *D. melanogaster* and humans were downloaded from NCBI (Accessed date: 19 September 2018, http://www.ncbi.nlm.nih.gov/). We used these protein sequences as queries to perform a BLASTP search against the silkworm genome database (Accessed date: 23 October 2019, https://silkdb.bioinfotoolkits.net/). The identification for each gene is listed in Figure 1B.

### 2.3. Plasmids

Expression constructs for polyamine pathway genes were amplified from the cDNA library of cultured silkworm cells using the primers listed in Table S1 and further cloned into an NcoI–XhoI/NotI site of pENTR11 (Invitrogen, Carlsbad, CA, USA) vector [30]. All plasmids were verified by sequencing. The pENTR11-BmODC, pENTR11-BmSAMDC, pENTR11-BmSPMS, pENTR11-BmSPDS, pENTR11-BmSSAT, pENTR11-BmAPAO, and pENTR11-BmSMO were respectively inserted into the expression vectors of pPBO_ie2GW (containing N-terminal EGFP tag) by gateway reaction to construct the expression plasmids [31].

### 2.4. EdU Staining

EdU staining was performed by using a commercial BeyoClick™ EdU-488 Kit (Beyotime, Shanghai, China). BmN cells were treated with spermidine at 10 μM or inhibitors at 50 μM (DFMO and MGBG mixtures in a molar ratio of 1:1) for 48 h and stained with 10 μM EdU for 2 h at 27 °C. The cells were fixed with stationary liquid for 15 min and then incubated with Click Additive Solution for 30 min behind the scenes. Subsequently, cells were stained with Hoechst 33,342 (Beyotime, Shanghai, China) and immersed in 1× PBS buffer. Finally, fluorescence signals were captured by a fluorescent microscope (Z16, Leica, Wetzlar, Germany). To quantify the EdU-positive cells (green), cells were analyzed by selecting three different fluorescent fields and counted by the counting function of Photoshop CS6 software (Adobe Systems Incorporated, San Jose, CA, USA).

### 2.5. Polyamine Measurements

Polyamine levels were measured by HPLC as described previously [32]. Briefly, sample pretreatment and derivatization: 1 × 10^7^ cells treated with inhibitors at 50 μM for 48 h were collected at 1000 rpm and immersed in 0.7 mL of 1× PBS buffer, ultrasonicated for 10 min, and centrifuged at 8000× *g* for 10 min. Then, 0.2 mL of supernatant were added into a brown centrifuge tube, and then 40 μL of 2 M NaOH, 60 μL saturated NaHCO_3_ and 0.2 mL of acetone solution of dansyl chloride (10 mg/mL) were added in order into a brown centrifuge tube (Light protection) (Axygen, San Francisco, CA, USA) and reacted in a water bath at 50 °C for 40 min in darkness. The reaction solution was cooled at room temperature, and 100 μL of ammonia were added into a brown centrifuge tube, and incubated for 30 min at room temperature. Finally, the total amount of 1 mL was filled using methanol and filtered by a pinhead filter. The experimental conditions were as follows: Chromatograph: agilent1100 high-performance liquid chromatograph with a wavelength of 254 nm; Column: Kromasil C18 reversed-phase column (250 mm × 4.6 mm, 5 μm); Column temperature: 30 °C; Flow rate: 1 mL/min; Injection volume: 10 μL; Mobile phase gradient: A, methanol; B, water. All assays were performed in triplicate.

### 2.6. Quantitative Real-Time PCR Analysis

To analyze the tissue expression characteristics of polyamine pathway genes and evaluate the mRNA levels of cell cycle-related genes after exogenous addition of spermidine or inhibitors, total RNA samples were prepared from BmN cells and silkworm tissues (Dazao strain) dissected on the third day of the fifth instar larvae of silkworm individuals by using Trizol reagent (Invitrogen, Carlsbad, CA, USA). According to the manufacturer’s protocol of the M-MLV Reverse Transcriptase Kit (Promega, Madison, WI, USA), 1 μg of total RNA was used for cDNA synthesis. qRT-PCR was performed with a SYBR Premix ExTaq Kit (Takara, Kyoto, Japan) and a qTower 2.2 Real-time PCR Detection System. The BmEif-4a gene was used as the internal control. All experiments were independently performed with three biological replicates, and the relative mRNA expression levels were calculated using the 2^−△△CT^ method. All primers used for qRT-PCR are listed in Table S1.

### 2.7. Subcellular Localization Assay

The analysis of fluorescently tagged proteins followed our previous protocol [30]. Briefly, cells were cultured on glass coverslips and transfected with 500 ng of expression plasmids. Then, 72 h post-transfection, cells were washed once with PBS and fixed with 4% paraformaldehyde in PBS for 10 min. The nuclei DNA were counterstained by 4′,6-diamidino-2-phenylindole (DAPI) (Invitrogen, Carlsbad, CA, USA). Fluorescence signals were captured by a fluorescent microscope (Z16, Leica, Wetzlar, Germany).

### 2.8. Flow Cytometry Analysis

Cell cycle distributions were monitored by flow cytometer analysis (CytoFLEX S, Beckman, Brea, CA, USA) of cellular DNA stained with propidium iodide (Sangon Biotech, Shanghai, China) according to the procedure described previously [30].

### 2.9. Statistical Analysis

Data are presented as the mean ± standard deviation (SD) of three independent biological replicates. Statistical significance (*p*-value) was analyzed by the Student’s *t*-test. Statistical significance is denoted as follows: * *p* < 0.05, ** *p* < 0.01, and *** *p* < 0.001.

## 3. Results

### 3.1. Identification and Expression Profiles of Polyamine Pathway Genes in Silkworm

Polyamines are widely found in prokaryotic and eukaryotic cells, and at least seven enzymes have been reported to be involved in the pathway of polyamine metabolism [7] (Figure 1A). In order to explore the function of this polyamine pathway in silkworm, we identified the genes in silkworm. We referred to proteins from the annotated species, including *D. melanogaster* and humans, and performed BLASTP analysis against the silkworm genome database, and named BmODC, BmSAMDC, BmSPDS, BmSPMS, BmSSAT, BmSMO, and BmAPAO (Figure 1B). Next, by qRT-PCR analysis, we analyzed the expression profiles of the polyamine pathway genes in silkworm tissues on the third day of the fifth instar larvae (Figure 1C), which showed that the polyamine pathway genes were expressed in all the examined tissues, although the expression levels of different genes in the same tissue were varied. The expression patterns of BmODC and BmAPAO were similar, both of which were highly expressed in Malpighian tubules. The expression pattern of BmSPDS was similar to BmSMO, and both were abundantly expressed in the fat body. The expression pattern of BmSPMS was similar to BmSSAT, both of which were predominantly expressed in the testis. These different expression profiles may indicate that polyamine pathway genes contribute to the regulation of the dynamic levels of polyamines in different tissues of the silkworm.

### 3.2. Subcellular Localizations of Polyamine Pathway Genes in Cultured Silkworm Cells

**Figure 2:**
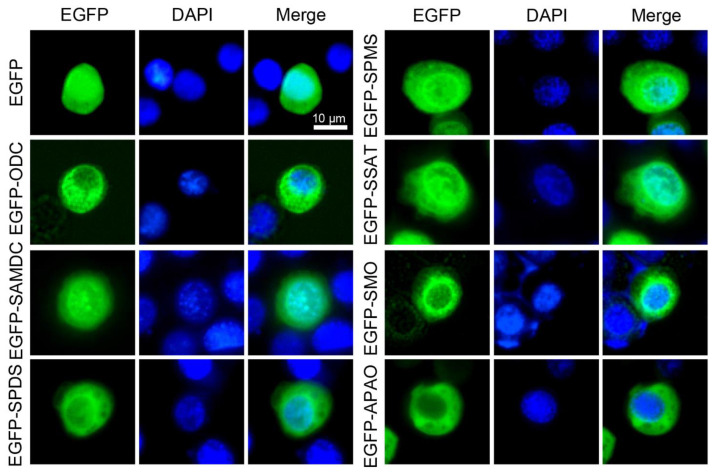
Subcellular localizations of polyamine pathway genes in cultured silkworm cells. Subcellular localization of transiently expressed EGFP-fused polyamine pathway genes in the silkworm BmN cells was determined by fluorescence (green) and the nuclei DNA was counterstained with DAPI (blue). Scale bar, 10 μm.

Subcellular localization of a target protein is frequently used to exhibit its function in cells. Enhanced green fluorescent protein (EGFP) expressed in cells is capable of producing green fluorescence when excited by illumination with ultraviolet light, which makes it an excellent reporter for in vivo observation [33]. Therefore, fusion expression of fluorescent protein with a target protein has been extensively used for direct visualization of target proteins in various cells and tissues by using confocal microscopy. To further analyze the potential functions of the polyamine pathway genes in cultured silkworm cells, polyamine pathway genes were constructed into the EGFP fusion expression vector, and then transfected into the silkworm cells for fluorescence observation. Subcellular localizations showed that BmODC, BmSAMDC, BmSPDS, BmSPMS, BmSSAT, BmSMO, and BmAPAO were predominantly localized to the cytoplasm and especially concentrated in the nuclear membrane as compared with the EGFP control (Figure 2). It was interesting that BmSAMDC and BmSSAT, both involved in the biosynthesis and metabolism of spermidine/spermine, respectively, also showed identical subcellular localization in the nucleolus, which may indicate their critical roles in regulating homeostasis of spermidine/spermine in whole cells. All these results revealed the specific localization of polyamine pathway genes in cultured silkworm cells.

### 3.3. Spermidine Supplementation Promotes DNA Replication in Cultured Silkworm Cells

**Figure 3:**
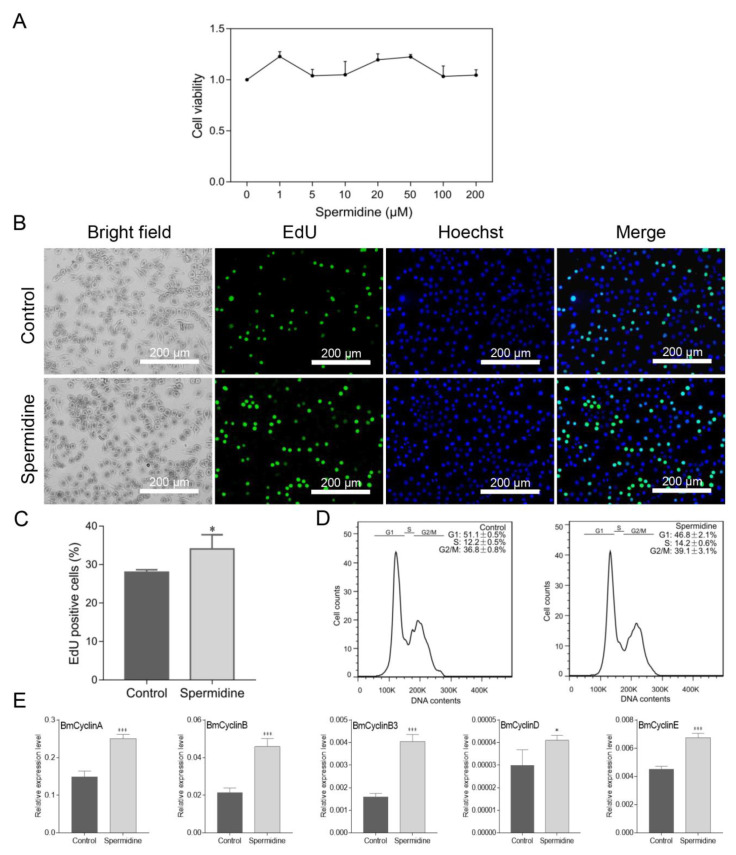
Spermidine supplementation promotes DNA replication in cultured silkworm cells. (**A**) Cytotoxicity test of different concentrations of spermidine on silkworm cells. (**B**) BeyoClick™ EdU-488 staining was used to detect DNA replication. Scale bar, 200 μm. (**C**) EdU-positive cells (green) were analyzed by selecting three different fluorescent fields and counted by Photoshop software. The data presented are the means ± SD (*n* = 3). (**D**) Cell cycle distribution was analyzed by using a flow cytometry and cells in G1, S, or G2/M were quantitated by FlowJo software. Three repeats were done for each treatment and the percentage of cells are the means ± SD (*n* = 3). (**E**) Expression of cell cycle-related genes was detected by qRT-PCR. For significant analysis: * *p* < 0.05 and *** *p* < 0.001.

Polyamines have been reported to promote cell cycle progression. To evaluate whether polyamines also contribute to the progression of silkworm cells, we added spermidine to the cells and measured its effects on the cell cycle. Firstly, the toxicity of spermidine was detected in silkworm cells and the results showed that spermidine, in the range of 0–200 μM, had no obvious toxicity effect on silkworm cells (Figure 3A). Then, we selected 10 μM of spermidine to treat the cells for further analysis. Ongoing DNA synthesis was detected in cells by using EdU staining, and it was shown that the treatment with spermidine clearly promoted DNA replication (Figure 3B). The proportional analysis showed that the number of EdU-stained cells was also significantly increased in spermidine-treated cells, which was consistent with the fluorescence observations of EdU staining (Figure 3C). Further, flow cytometry analysis was used to examine the phase of the cell cycle after spermidine treatment. It was shown that spermidine was able to increase the percentage of cells at the S and G2/M phases and decrease G1 phase, which suggests that spermidine may promote cell cycle progression (Figure 3D). Interestingly, spermidine supplementation markedly induced the expression of cell cycle-related genes, such as BmCyclinA, BmCyclinB, BmCyclinB3, BmCyclinD, and BmCyclinE (Figure 3E). Taken together, it was shown that spermidine supplementation in silkworm cells can promote DNA replication and cell cycle progression by inducing the expression of cell cycle genes.

### 3.4. Inhibition of Polyamine Pathway Activity Reduces Polyamine Levels of Cultured Silkworm Cells

**Figure 4:**
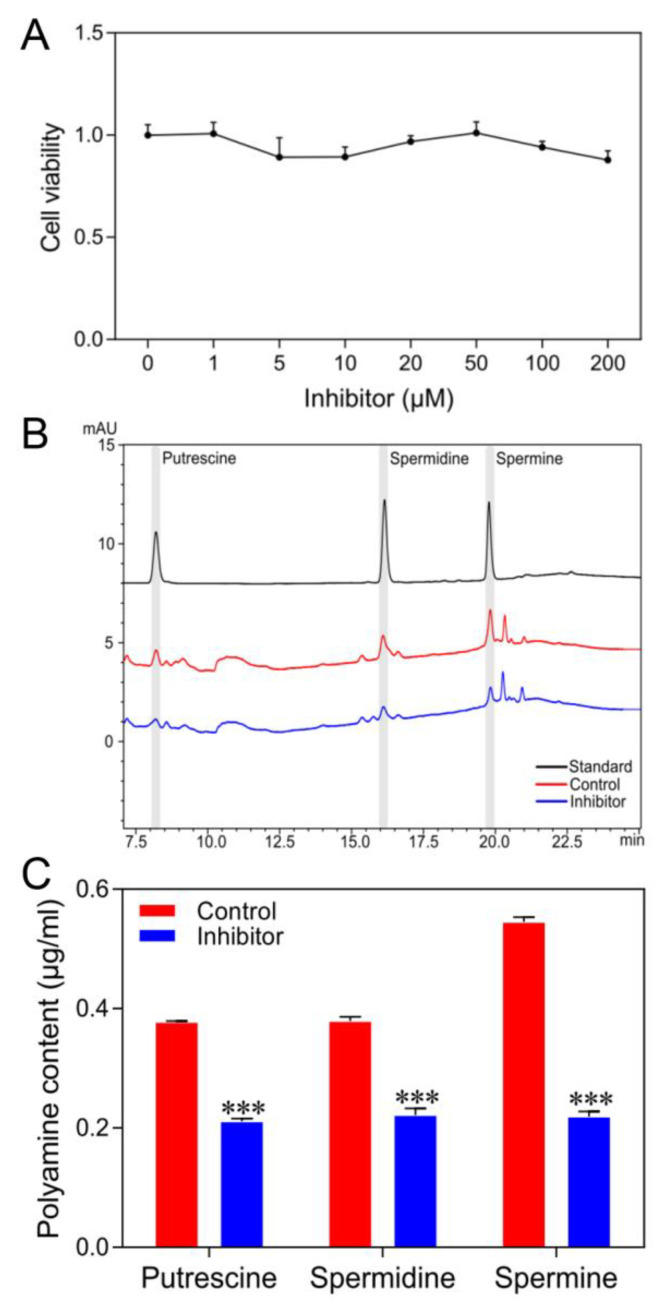
Inhibition of polyamine pathway activity decreases the levels of polyamines in cultured silkworm cells. (**A**) Cytotoxicity test of different concentrations of spermidine on the silkworm cells. (**B**) The standards of various polyamines and intracellular polyamines were determined by HPLC and the representative figure is shown. (**C**) Three independent experiments for polyamine content were analyzed, and the data presented are the means ± SD (*n* = 3). For significant analysis: *** *p* < 0.001.

To further explore whether a decrease of intracellular polyamine levels would affect the cell cycle activity of silkworm cells, ODC inhibitor (DFMO) and SAMDC inhibitor (MGBG) were used to inhibit the activity of polyamine biosynthesis. To determine the toxicity of inhibitors on cultured silkworm cells, first, we treated the cells with DFMO and MGBG mixed at a molar ratio of 1:1. The results showed that treatment with the inhibitors had no obvious toxicity in the range of 0–200 μM on cells (Figure 4A). Then, cells were treated with 50 μM concentrations of inhibitors for further experiments. After the treatment with the inhibitors, we collected the cells and measured different polyamine levels by HPLC analysis. As shown in Figure 4B,C, it clearly showed that the levels of putrescine, spermidine, and spermine were significantly decreased in cells. These results indicated that the treatment with inhibitors successfully reduces polyamine levels in silkworm cells.

### 3.5. Inhibition of Polyamine Pathway Activity Decreases DNA Replication in Cultured Silkworm Cells

**Figure 5:**
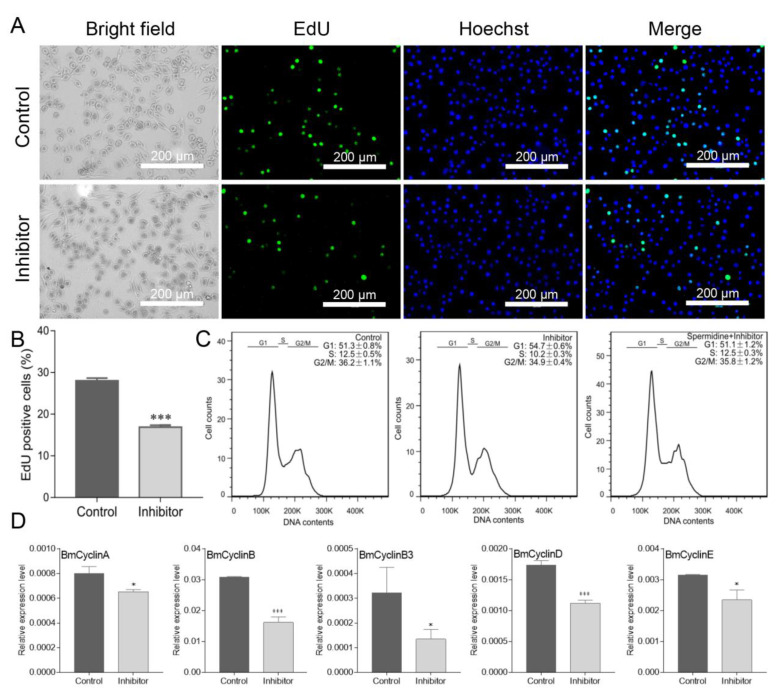
Inhibition of polyamine pathway activity decreased DNA replication in cultured silkworm cells. (**A**) BeyoClick™ EdU-488 staining was used to detect DNA replication. Scale bar, 200 μm. (**B**) EdU-positive cells (green) were analyzed by selecting three different fluorescent fields and counted by Photoshop software. The data presented are the means ± SD (*n* = 3). (**C**) Cell cycle distribution was analyzed by using a flow cytometry and cells in G1, S, or G2/M were quantitated by FlowJo software. Three repeats were done for each treatment and the percentage of cells are the means ± SD (*n* = 3). (**D**) Expression of cell cycle-related genes was detected by qRT-PCR. For significant analysis: * *p* < 0.05 and *** *p* < 0.001.

Next, we investigated DNA replication ability by EdU staining when the levels of intracellular polyamines were reduced by treatment with inhibitors. The result showed that the DNA replication ability was attenuated (Figure 5A), and the proportion of the EdU-positive cells was also significantly decreased (Figure 5B). The effect of inhibitors on the progression of the cell cycle was also evaluated. As shown in Figure 5C, a delay of the transition from G1 to S phase was observed, and the percentage of cells at the G1 phase was higher in cells treated by inhibitors than that in control cells. Interestingly, the G1 arrest of cells by inhibitors could be partially recovered by the addition of spermidine (Figure 5C), which further confirmed the role of spermidine in regulating cell cycle progression. In contrast to spermidine supplementation, the qRT-PCR analysis of the expression for cell cycle genes revealed that the treatment with inhibitors decreased the expression of all the genes (Figure 5D). Taken together, these results showed that the dynamic levels of polyamines in cultured silkworm cells play important roles in regulating cell cycle progression, and the traditional inhibitors of the polyamine pathway can be used to investigate polyamine functions in the silkworm.

## 4. Discussion

In the current study, we identified the anabolic and catabolic enzymes that are involved in the polyamine biosynthetic pathway in the silkworm, and analyzed their expression characteristics in different tissues, as well as their subcellular localization patterns in cultured silkworm cells. Moreover, we investigated the role of the polyamine pathway on DNA replication and cell cycle progression by the exogenous additions of spermidine and inhibitors. The obtained data expanded the functional insights of polyamines in the lepidopteran model insect.

Biosynthesis of polyamines is regulated by a series of enzymes [7]. For example, ODC and APAO are involved in the synthesis of putrescine, SPDS and SMO co-regulate the synthesis of spermidine, while SPMS and SSAT regulate the homeostasis of spermidine and spermine. It was speculated that the enzymes regulating the synthesis of the same substance may have similar expression characteristics in a given tissue. Therefore, we analyzed the tissue expression profiles of polyamine pathway genes in the silkworm individuate. Although the tissue expression characteristics showed that polyamine pathway genes were expressed in all analyzed silkworm tissues, their expression levels were varied. It was interesting that the expression patterns of BmODC/BmAPAO, BmSPDS/BmSMO, and BmSPMS/BmSSAT are similar in different tissues, and have very high expression in a specific tissue. These observations showed that the varied expression abundances of polyamine pathway genes in different tissues contribute to different polyamine levels, and suggested that different levels of polyamines are important in different tissues.

Polyamines are involved in a number of biological processes, including promoting cell proliferation and cell cycle progression [6,13]. Consistent with this, exogenous addition of spermidine promotes DNA replication and cell cycle progression of cultured silkworm cells. Meanwhile, treatment of cells with polyamine pathway inhibitors DFMO and MGBG prevents DNA replication and the transition from G1 to S phase is also delayed, and importantly, the levels of putrescine, spermidine, and spermine are significantly decreased by inhibitors. Therefore, the efficacy of DFMO and MGBG for inhibiting polyamine levels in silkworm cells provides the potential to investigate polyamine functions by inhibitor treatment in silkworm individuals.

Furthermore, we found that the cell cycle-related genes, including BmCyclinA, BmCyclinB, BmCyclinB3, BmCyclinD, and BmCyclinE, were all upregulated by spermidine supplementation and downregulated by inhibitor treatment, respectively, which implied that spermidine may affect cell cycle progression by participating in the regulation of cell cycle gene expression. This is consistent with the view that polyamines can regulate gene expression by binding to negatively charged nucleic acids and altering chromatin and RNA structure or increasing translation efficiency [34]. Whether spermidine can directly regulate the cell cycle gene expression or not needs to be explored in the future.

Indeed, it has been reported that silkworm fed with spermidine-treated leaves enhanced the expression of BmFib-H, a gene encoding for fibroin heavy chain that is the largest and most abundant silk protein in the posterior silk glands of the silkworm [35]. Moreover, the quantity and quality of silk were improved by the spermidine treatment [27,28], which indicates the potential application of polyamines for better silk production. We also examined the silk properties after the injection of spermidine into the fifth instar larva of the silkworm strain stocked in our laboratory; however, no significant changes were observed in the cocoon weight and shell ratio of the spermidine-treated silkworm as compared with the control (data not shown). The difference in our findings might be due to the different silkworm strain or different treatment of spermidine supplement. However, we will detect whether spermidine could enhance the expression of silk proteins and regulate endoreplication of silk gland cells in the next work. Because of the critical role of ODC, a rate-limiting enzyme in polyamine synthesis, we are currently establishing the stable expression of ODC in the silkworm in order to increase the levels of polyamines in the silk glands. It is expected that this strategy could provide us novel insights into regulating the expressions of silk proteins and increasing the yields of silk in silkworm.

## 5. Conclusions

In summary, this study is the first to analyze the polyamine biosynthetic pathway genes in the lepidopteran silkworm and to investigate the role of spermidine on DNA replication and cell cycle progression of cultured silkworm cells, which demonstrated the conserved function of the polyamine pathway among species. Our data also supported that commercial polyamines and inhibitors can be used to investigate the roles of the polyamine pathway in silkworm cells and individuals. Future studies need to clarify the molecular mechanism by which polyamines regulate the expression of target genes in silkworm.

## Data Availability

Data are contained within the article and Supplementary Materials.

## Supplementary Materials

The following are available online at https://www.mdpi.com/article/10.3390/insects12070624/s1, Table S1. List of primers used in this study.
