# Supplementary material for: Function of Polyamines in Regulating Cell Cycle Progression of Cultured Silkworm Cells
Source: Insects. 2021 Jul 8;12(7):624. doi: 10.3390/insects12070624 (PMC8306941; doi:10.3390/insects12070624)
Supplement: Supplementary file 1 [file insects-12-00624-s001.zip › insects-1248626-supplementary.pdf]

Supplementary Information

Table S1. List of primers used in this study.

Gene name	Sequence (5'-3')
BmODC-atg	AAGGTTGTGGAGGAACAACGCATCAGA
BmODC-R-XhoI	CCCTCGAGTCACTTGAGCGCACACTCGACG
BmSAMDC-atg	GCTGAAACGGAGATAATATCAAATAG
BmSAMDC-R-XhoI	CCCTCGAGTCAGCTCGGGAACTTGCAGTACA
BmSPDS-atg	GACAAACTAAAAACCAACTGGTTTACGG
BmSPDS-R-XhoI	CCCTCGAGTCAAGCCAGTTTATATTTTAC
BmSPMS-atg	TCAGTTCACAATATCTTATTGGATTTCTCG
BmSPMS-R-NotI	TTGCGGCCGCTTAATCATCTCCAGTTCCTTTG
BmSSAT-atg	TCTAACAAATATAAAAGCGGTGAAG
BmSSAT-R-XhoI	CCCTCGAGCTAGCCATCTTTGGCAAAGTCA
BmAPAO-atg	GGTGACGAAAATTTAGGTTCCAGTGAAAAATG
BmAPAO-R-XhoI	CCCTCGAGTCACCCTTCCAGCTGTATTCCTTG
BmSMO-atg	GACGTTATCGTAATAGGCTGCG
BmSMO-R-XhoI	CCCTCGAGTTATTTTTGGTTAGGTTTGACGTTTG
BmODC-F	TGGAGGAACAACGCATCAGAG
BmODC-R	CAGGGTGGTCAACAGCAACTT
BmSPDS-F	TGCTGCCACAAAAATCCA
BmSPDS-R	TTCAAAACCGTCACCCAC
BmSAMDC-F	CTGCGGCTACTCTATGAA
BmSAMDC-R	GATGGTGAGCACGAACTT
BmSPMS-F	ACATCCCCATCTCCGACACC
BmSPMS-R	GGCACGAACGCCATGCTCCT
BmSSAT-F	TGAGAATGAATGAGCGGC
BmSSAT-R	TAAAACGAACAAGCAGGA
BmAPAO-F	AGATTGCGGAGGTGGTGA
BmAPAO-R	TGGCTGCGAGCGTGTAGG
BmSMO-F	TTTTGAGGTCACAGTGGG
BmSMO-R	ATTGCCGTGGTAGATAGG
BmeIF-4a-F	TTCGTACTGGCTCTTCTCGT
BmeIF-4a-R	CAAAGTTGATAGCAATTCCCT

BmCyclinA-F	TGCGGAGCTGTGCCTACT
BmCyclinA-R	ATTGCGGGTTGACCTTGC
BmCyclinB-F	ATTGACAGATATTTGCAGGTTG
BmCyclinB-R	GACAGAAGCCCAGTTTGC
BmCyclinB3-F	GCTCGTGTATCAATGCCTAC
BmCyclinB3-R	GCTAATGCAATCGGGAGA
BmCyclinD-F	GTTTCGTCAGTGTCATCCG
BmCyclinD-R	GTTAAGGTGAGGGCGTGT
BmCyclinE-F	GTCCCTTGCCTGGATTGT
BmCyclinE-R	ATTGCCGTGGTAGATAGG
